# Changes in Caregiver Personal Support Networks: Gender Differences and Effects on Health (CUIDAR-SE Study)

**DOI:** 10.3390/ijerph182111723

**Published:** 2021-11-08

**Authors:** María Nieves Rodríguez-Madrid, María del Río-Lozano, Rosario Fernández-Peña, María del Mar García-Calvente

**Affiliations:** 1 Fundación para la Investigación Biosanitaria de Andalucía Oriental (FIBAO), 18012 Granada, Spain; nrodriguezmadrid@yahoo.com; 2 Escuela Andaluza de Salud Pública (EASP), 18080 Granada, Spain; maria.rio.easp@juntadeandalucia.es (M.d.R.-L.); mariadelmar.garcia.easp@juntadeandalucia.es (M.d.M.G.-C.); 3 Instituto de Investigación Biosanitaria de Granada ibs. Granada, 18012 Granada, Spain; 4 Faculty of Nursing, University of Cantabria, 39008 Santander, Spain; 5 IDIVAL Nursing Research Group, 39011 Santander, Spain; 6 SALBIS Research Group, University of León, 24400 León, Spain

**Keywords:** informal care, gender perspective, social network analysis, personal support networks, longitudinal study

## Abstract

Technological changes have led to important advances in medical diagnoses and treatments that prolong the informal care process. Support from the personal network of informal caregivers is an undervalued resource and the changes that have occurred over time are unknown. The aim of this study was to analyze the changes in personal network support among informal caregivers and to examine the effect of these changes on self-perceived caregiver health, with a focus on differences between men and women and caregivers with high and low levels of burden We also investigated caregiver perceptions and explanations of changes to their support network (losses and additions and no change). Using a mixed-methods approach, data were obtained from 32 caregivers that were intentionally selected in Spain, who were interviewed twice with a one-year interval. In the quantitative phase, personal networks analysis was performed with Egonet software, which obtained data on the composition and functional content in social support from 1600 personal relationships (25 alters for each ego in the two waves). In the qualitative phase, semi-structured interviews were conducted in the two waves with a guide in order to explore the changes in informal support resources over time. The selected men with high levels of burden pointed out a loss of network support with more discouraging reports compared with the low-burden male caregivers. Furthermore, the selected women with low burden levels mentioned losses too; however, their reports were more positive. Women reported improved health, especially those with low burden scores in the first wave and those who did not lose support. Caregivers with a high initial burden and who lost support reported worse health, particularly men and women with a strong sense of duty toward care. Social support from personal networks is important for caregiver health and its effects are influenced by gender roles. Our findings could help by improving the relational and social capital of informal caregivers and adapting them to the new needs of formal home care systems.

## 1. Introduction

All people need to be cared for at some point in their lives, but particularly during childhood, old age, and periods of chronic illness or disability. Most informal care takes place in the home and is usually provided for by people from the care recipient’s immediate environment, mainly women [1]. Informal care is unpaid [2]. In Spain, as in other southern European countries, the family and the close social circle play an important role in the care of dependent people, and this informal care is characterized by a high intensity. More than 50% of the people who provide informal care do so for more than 20 h per week [3]. The Spanish Dependency Law [4], launched in 2007, was a major step forward in improving the social recognition of caregivers. This law gave rise to the current System of Autonomy and Care for Dependency. This system provides a set of services and benefits that are aimed at promoting personal autonomy, as well as the protection and care of people through accredited public and private concerted services [5].

There is evidence of gender inequalities in the care process [6], not only in terms of the impact of caregiving on caregiver burden and health [1,7,8,9] but also in the different ways that men and women perceive and experience caring, cope with its obligations [7], and organize their resources. Underlying these differences are deep-rooted gender norms that have historically assigned women the role of the caregiver [10].

Little has been published on changes to the relational environment of male and female caregivers, although it was shown that caregiving can result in considerable personal network turnover [11]. This is because it involves not only action and dedication but also concern, responsibility, and a willingness to help (caring for vs. caring about). Both of these dimensions encompass tasks that transcend the scope of specific diseases to move into the realm of everyday life, where everybody needs to be taken care of and, at the same time, is capable of taking care of others [12]. These dimensions are also closely linked to the ethics of caregiving and gendered notions that caregiving is essentially a feminine job [13].

Studying informal care from the perspective of time and relational dynamics requires viewing caregiving as a process, with a starting point and a non-uniform trajectory that, over time, can result in a difficult time for caregivers. The literature shows that caregivers who enjoy informal support from their social networks have lower levels of caregiver burden and better self-perceived health [14,15,16]. It is also important to remember that most informal care takes place between people who are socially related [17].

Social support is considered to be a resource that is derived from the relationships that make up a social network, which is defined as a “set of actors and the ties among them” [18]. From a relational approach, social support is one of the areas of application of the methodology that is based on social network analysis (SNA) [19], which is a research method that examines the interactions between individuals, groups, and organizations. Personal network analysis [20], also named egocentric networks in the literature, is an approach that is based on SNA and involves a subset of the broader concept of egocentric networks. This approach targets the relationships surrounding individuals in all the social environments to which they belong (e.g., family, co-workers, and neighbors) [21]. It is a methodology that is used in other social support studies [22,23,24]. Networks are the means by which social support is distributed and exchanged [25], and this support can take different forms (e.g., material or emotional). Social support networks are small but proactive and they give rise to even smaller care networks. Accordingly, a caregiver with a wide social network will not necessarily receive greater caregiving support [26].

While there is evidence that caregiving can alter personal networks and relationships [11,27], few longitudinal studies have analyzed the network turnover among male and female caregivers. There is also evidence that women and men approach caregiving differently. In a previous cross-sectional study by our group, we found that informal male caregivers received more help from members of their network with specific caregiving tasks, such as personal care and household chores. Female caregivers, by contrast, received more help with activities outside the home and physical mobility tasks [24].

Studying social support from the perspective of personal networks helps to uncover the social structures underlying the different relationships that male and female caregivers forge during the care process. Qualitative studies can deepen our understanding of how caregivers perceive changes in social support and how these changes affect their health. They can also help to capture the emotional and affective components of caring (caring about).

The aim of this study was to analyze the changes to caregiver personal support networks and investigate how these changes affected self-perceived caregiver health, with a focus on the differences between men and women and caregivers with high and low levels of burden. We also sought to gain insights into caregiver perceptions and explanations of involvement or non-involvement by members of their personal network in the process of care. Analyses of caregiver support networks beyond the perspectives of primary caregivers are essential for providing a better understanding of how the social environment responds to the demands of care.

## 2. Materials and Methods

### 2.1. Design

We performed a two-wave mixed-methods longitudinal personal network analysis [20] that combined quantitative data on the social support function of networks and qualitative data that was obtained via semi-structured interviews.

### 2.2. Intentional Sample

The study population was formed by male and female informal caregivers aged 18 years or older living in the province of Granada (Spain) who were providing unpaid care to a family member or a person from their personal settings. The intentional sampling was carried out based on the database of the CUIDAR-SE study [8], which is a larger study that has 404 registered informal caregivers from the Granada Health District. In the first wave of the study, we selected 50 caregivers on a purposive basis, divided into two groups: 25 men and 25 women. The goal was to achieve maximum heterogeneity within each of the groups in terms of age, level of education, place of residence (urban vs. rural), time spent caregiving, and level of caregiver burden, as assessed using the Zarit Burden Interview [28]. Thirty-two of the 50 people from the first wave completed the second wave. The reasons for loss to follow-up were the death of the care recipient (*n* = 9) and refusal to continue (*n* = 9). The results presented reflect changes in the personal networks of 32 caregivers (egos) who completed the follow-up phase. There were 16 men (egos) and 16 women (egos), and 800 people who formed part of their social settings (alters).

### 2.3. Data Collection

The data were collected by the same researcher between September 2017 and March 2018 (first wave) and November 2018 and March 2019 (second wave). The data collection included the quantitative phase, which involved personal network data collection, and the qualitative phase, which involved semi-structured interviews. The duration of the interviews ranged between 60 and 90 min. The participants chose to do the interview in the home in which they were providing care, and every effort was taken to ensure their comfort and privacy. The interviews were tape-recorded with prior permission from the caregivers.

In the first wave, EgoNet open-source software https://sourceforge.net/projects/egonet/ (accessed on 15 March 2019) was used to collect each participant’s personal network data. Personal network data were collected through four modules:

1st module: Information on the sociodemographic characteristics of each participant (ego).
2nd module: A name generation question that focused on identifying the people in their network (alters) belonging to the different areas of social life in which they are embedded (family, friends, co-workers, and neighbors, among others). Participants were asked to list a fixed number of 25 alters [29,30].
3rd module: Variables regarding the composition of the personal network (alter char-acteristics) and variables related to social support (social support function).
4th module: Informants were asked about the relationship between possible pairs of actors among the nominated contacts.

In addition, once the information from the four modules had been collected, we obtained a graph visualization of the personal network [31] with the aim to obtain qualitative information about their social and relational environment, as well as the social support received.

The graphs that were obtained showed the members of each personal network (nodes) and their relationships (lines). Composition and functional variables, such as gender and type of support offered, were depicted by node colors, sizes, and shapes. Each ego identified and described the different groups and explained the meaning that the relationship had for them. Finally, a semi-structured interview was held to enable a qualitative analysis of the caregiver perceptions and explanations of their relational environment and the effects of support on their health (Appendix A).

In the second wave, a second semi-structured interview was held at the home of the care recipient to update the personal network information that was collected a year earlier using a paper copy of the original graph. Using the EgoNet software and the same ad hoc questionnaire as in the first wave, each ego updated their network by adding or removing alters as appropriate and modifying the personal and support characteristics of contacts who remained in the network. This procedure for collecting personal network data in longitudinal studies has been used by other authors [32]. New graphs were obtained using the same legend as in the first wave and, supported by the visualization of their network graph, semi-structured interviews were used to explore the changes that had occurred during the year (Appendix A).

### 2.4. Data Analysis

We compared the compositional data and functional content in the social support of the personal networks of the men and women that were selected in two different waves. Data from 800 alters were compared (400 in the women’s networks and 400 in the men’s networks); therefore, we analyzed the evolution of 1600 relationships. The method that was used to quantitatively analyze the relationships between multiple variables was cross-tabulation tables. Descriptive statistics presented as numbers and percentages were calculated in the statistical program SPSS (IBM SPSS Statistics for Windows, Version 25.0. Armonk, NY, USA) and the results from the two waves were compared and expressed as percentage differences. The dimensions of the social support function within the network were as follows: help with specific caregiving tasks and emotional support. The variables that were used to measure the support function were the number of alters who did not help with specific caregiving tasks, number of alters who helped with specific caregiving tasks (categorized as personal care, physical mobility, household chores, nursing-type tasks, supervision/company keeping, activities outside the home), frequency of help provided, changes over the past year, and the number of alters offering emotional support. These variables are in rows. The columns were the caregiver gender and level of caregiver burden on the Zarit scale, dichotomized as none or low (score < 55) vs. high (score ≥ 56).

In the qualitative phase, content and discourse analyses were accomplished. The analyzed categories were perceived changes in support, explanations of why some people provided help and others did not, and the impact of these changes on self-perceived health. We also analyzed the differences in the use of language by men and women to capture practices, meanings, and social contexts of the relationships within the networks. Comparison of discourses from the first and second waves provided insights into how individual caregivers interpreted changes to their personal networks and produced a biographic narrative account of the different caregiving situations that provided insights into the dynamics of the personal network.

The qualitative analysis was performed from a reflexive position and consisted of the following steps: (a) verbatim transcription of the interviews, (b) close reading of the transcripts, (c) formulation and recording of the analytical intuitions, (d) interpretation of discursive positions according to the characteristics of each caregiver, (e) analysis of the symbolic configurations and semantic spaces within and between the different texts, and (f) synthesis of the main findings in light of the study objectives.

The analysis was conducted independently by two members of the research team, whose findings were then triangulated, discussed, and jointly interpreted as part of the data validation and quality control process.

### 2.5. Ethical Considerations

The study was approved by the Biomedical Research Ethics Committee of Andalusia. The participants were informed orally and in writing about the study objectives and provided signed informed consent.

## 3. Results

### 3.1. Description of the Intentional Sample

The sociodemographic and caregiving characteristic of the 16 men and 16 women selected who participated in the study are summarized in Table 1. Caregivers with a high level of caregiver burden, aged <65 years, living in an urban area, and with a secondary or tertiary level of education were more common. More than twice as many men as women were looking after their partners, and more than three times as many daughters as sons were looking after their mother or both parents.

### 3.2. Changes in Network Support According to Caregiver Gender and Level of Burden

The selected men in our study reported a slightly greater loss of help from their network with specific everyday caregiving tasks than women between waves 1 and 2 (Table 2). Women, by contrast, noted that they lost more emotional support in relation to both caregiving and non-caregiving matters. Help with household chores remained stable among men and women, and women pointed out a greater loss of help with physical mobility tasks than men.

Changes in support according to gender and level of caregiver burden are shown in Table 3. The men of this selection who were severely burdened experienced a greater loss of help with specific caregiving tasks but gained emotional support in relation to caregiving matters. In the reports of these men, a certain disappointment, displeasure, and discouragement were present when expressing the closeness or emotional proximity of the members of their network, even if they maintained the same response between waves 1 and 2. Men with a low burden, by contrast, experienced no changes in help with specific caregiving tasks but did lose emotional support. The use of the expression “*everything remains the same*” was frequently repeated. Selected women’s experiences were different. Loss of help with specific caregiving tasks was the lowest among severely burdened women, but they also experienced the greatest gains in emotional support for matters that were not related to caregiving. These women did not want to emotionally involve members of personal support networks in caregiving, especially if they were male children. Women of our selection with a low burden experienced a greater loss of help with specific caregiving tasks than those with a high burden. However, their reports reflected acceptance and gratitude when they referred to these types of support.

### 3.3. Caregiver Perceptions and Explanations of Support Received and Not Received from Their Personal Network

Both male and female selected caregivers saw geographical distance as a key determinant of support provision. They felt they could ask for help from a person who lived nearby but mentioned distance as a reason for not receiving help. The frequency and intensity of help required increased with caregiver burden and/or care recipient dependency, and the geographic proximity of people in the network was highly valued. Caregivers who had been providing care for more than 10 years or who had chronic health problems viewed their situation as persisting into the medium or long term, and therefore perceived distance as a key factor regarding care arrangements. Most of the solutions they proposed involved moving closer to the family network.

Children living in the home were asked more often to help with caregiving tasks as they grew older and were assigned more responsibility when they were daughters or only sons. Both male and female caregivers mentioned that a worsening of their health led to a redistribution of support in the form of more help from secondary caregivers that were already in the network.

Women reported that they did not ask for help because they “didn’t need it” or because it “wasn’t necessary” more than men. They felt capable of shouldering the burden themselves, as long as it did not become too great, even at an advanced age. When they did ask for help, it was in exceptional or serious circumstances. When faced with an increase in burden or a deterioration of health, women reconsidered their situation, switched strategies, and delegated more responsibility to members of their network more than men. They were also more open about describing what had influenced their decisions to “look after” the person in their care or to “take on” this responsibility. Their biographical accounts show that caregiving was part of the socialization process of women in their families. Because of these experiences, they asked for less help from their network and showed appreciation when they received it and resignation when they did not. By contrast, men who cared for a member of their family other than their partner were either the only child or had taken over from a female primary caregiver whose situation had changed (mainly a worsening of health). A range of socio-occupational and family-related reasons (paid employment, working hours, being an informal caregiver) was also mentioned to explain why most of the alters in the network did not participate in caregiving. Both genders reported that the women in their personal networks were already looking after young children or older people.

### 3.4. Impact of Personal Network Support on Caregiver Health

The impact of support on self-perceived health varied according to caregiver gender and burden. Caregivers of both genders, but women in particular, reported improved health or no changes when they had low burden scores in the first wave or had gained support from their network. Caregivers with a low burden and who had gained or maintained support showed more self-control, inner strength, and acceptance. They largely attributed their physical health problems to age. Women mentioned the need to “look after themselves” to be able to provide better care and to plan their caregiving duties more efficiently more than men. Nonetheless, caregivers with a low level of burden also mentioned an improvement in health when their overall workload decreased, even if they had lost support. In other words, support did not have such a decisive effect on self-perceived caregiver health in the presence of key events, such as an improvement in the dependency situation, changes in overall workload (paid or unpaid), or the death of another person they were caring for.

Caregivers who were already severely burdened and who lost support over the year reported a deterioration in health, particularly when they were men. More men than women reported difficulties with everyday caregiving tasks and stress-related psychological problems. During the first wave, more women stated that they had assumed the role of caregiver out of obligation and that it was a responsibility that largely affected their everyday decisions and health. In the second wave, they described situations that had exacerbated existing health problems, such as delayed operations or the renouncement of leisure activities. In the first wave, male caregivers referred more to emotional exhaustion and/or the long time for which they had been providing care, while in the second wave, they were more likely to mention feelings of anxiety and sadness, or behaviors involving escape strategies, self-distraction, and social isolation.

### 3.5. Four Case Studies

The next section shows four personal network graphs illustrating changes to support received with specific caregiving tasks according to caregiver gender and burden. Green circles and squares correspond to alters who helped with specific caregiving tasks, while black circles and squares correspond to those who did not. Women are shown as circles and men as squares. Large nodes indicate an increase in support, while small nodes indicate a decrease. In addition, each graph is accompanied by a selection of verbatim quotes (translated from Spanish to English) that illustrate the categories obtained in the analysis of the qualitative data.
Case 1: Man, 59 years old with a high caregiver burden. He had been looking after his mother and mentally disabled brother for 4 years.
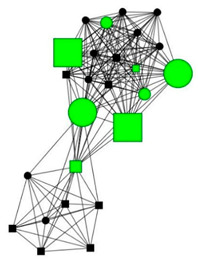

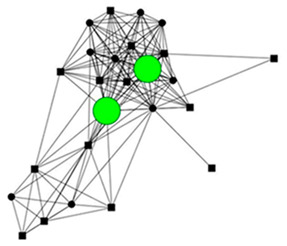
Case 1—First waveCase 1—Second wave*Categories**Perceived changes in support**“I feel so alone. During the holidays, I ask my brothers to give me a week’s rest to switch off… and they answer; “what we need to do is send them to a home, they’ll be looked after better there.”**“Every year, as they see my mother becoming weaker and weaker, they remove themselves more from the picture, they don’t want to know.”**Explanations of why**“They don’t help me because they don’t want to know anything about anyone…they don’t ask me for any explanations.”**“Some because they’ve moved away to study or work and others because they’ve become fed up. And they seem more distant to me…no company is better than bad company. And the new ones, they are people who see me and try to cheer me up […] People who show concern towards me are a great help. It’s not that they physically help me, it’s that they listen to me.”**Impact on health**“Because I’ve often felt overwhelmed because I’m depressed…I have arthritis, I have everything under the sun. [The man interviewed replicate with resignation the regular conversation than usually has with their other brothers]—I need help, can you come.—Relax, relax, they say.”**“I took time off work for the mental health service. [when I was not caring] because I’ve been a person who’s come and gone…, I’ve done everything.”*
Case 2: Man, 54 years old with a low caregiver burden. He had been looking after his partner, who had chronic fatigue syndrome and fibromyalgia, for 13 years.
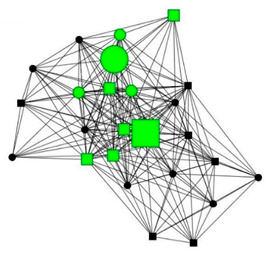

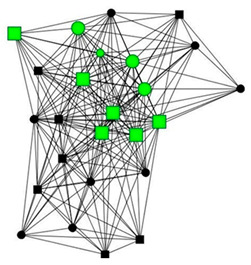
Case 2—First waveCase 2—Second wave*Categories**Perceived changes in support**“If you had done this (the interview) 8 years ago, I would have been better, there would be more people in my network. […] Everything was rearranged. The life we had before wasn’t the same. You accept it and adapt because you have no choice… We can seen the way forward, and with the help of a lot of people, who are there unconditionally.”**“It’s more or less the same, everything’s the same, yes, the affection is the same but we see each other less now.”**Explanations of why**“they don’t help me because… there are lots of reasons…these are friends, very good friends, it was my wife who distanced herself more from them.”**“The neighbor helped more before last year and this year she’s helped even less… they’ve distanced themselves because they are looking after her mother and she doesn’t have so much time anymore.”**Impact on health**“I feel a little more down now. Although I don’t think it’s related (to the caregiving), it’s not the same being 30 as 54.”**“yes, in the sense of the caregiver, it’s exhausting, exhausting, because psychologically the caregiver, has a lot of ups and downs and that’s what’s worse for me, largely in the emotional sense (…) I felt much better last year and the year before.”*
Case 3: Woman, 67 years old with a high caregiver burden. She had been looking after her mother for 17 years.
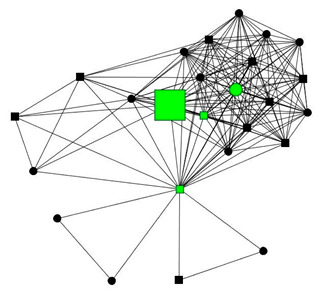

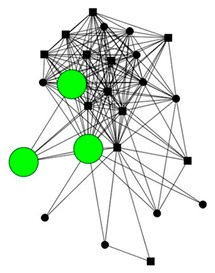
Case 3—First waveCase 3—Second wave*Categories**Perceived changes in support**“Yes, moral support as much as you want, but nothing else.”**“I’ve still got my friends you see, what’s more, I’ve got more, more, I always keep people in my life. […] My son used to come. He doesn’t any more. Neither does my other son, or my daughter in law, nothing…They have never done anything. […] My mother has gone to a flat that I’ve rented in the village and I’ve hired live-in help […] My sister and myself are looking after everything.”**Explanations of why**“everyone has their own life and I took it on myself to bring my mother home because she was living in a flat with a girl. I did the same with my mother in law and her sister. I’ve had a mini-nursing home here.”**“my brothers all have their own problems. (…) I was born feeling that I was my brothers’ mother and everyone’s mother. Why? Because I’ve been a mother to everyone since I was 13 and nobody can take that feeling away from you. Or that obligation, because you are born prepared to serve everyone’s needs. And it’s very difficult to undo that.”**Impact on health**“I’m broken…, the tendons in my shoulder are torn, they operated on me and they tore again (for carrying my mother) […] there are days when I feel really bad…, others when I’m OK, but my health has deteriorated by about 40%. Oh yes, it has definitely deteriorated!”**“I’m in quite a bad state (fucked) but that’s how things are. I had back pain this year and when my husband saw me walking with a stick… And he said to me;…, what sort of life is this…?! So, that got the ball rolling…, the house feels more lonely …, but on the other hand, I’m sleeping more peacefully now… I’ve gained in quality of life, freedom of movement.”*
Case 4: Women, 57 years old with a low caregiver burden. She had been looking after her partner, who had reduced mobility due to paralysis, for 20 years.
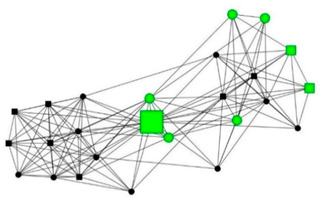

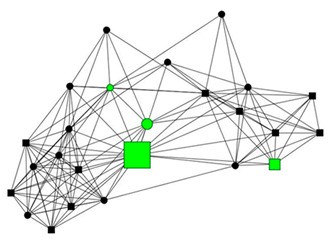
Case 4—First waveCase 4—Second wave*Categories**Perceived changes in support**“My son helps us, move him, wash him, we take him to church, to the library. He has been helping more and more, because he’s growing, before he couldn’t move the wheelchair.”**“We removed them because they have died, the others are from the social services…the women who came to help me bathe my husband were from a program that no longer exists…we haven’t had any contact with them since. […] the other ones, I see them more some evenings, but we still see each other.”**Explanations of why**“People who don’t help me at home, but they help us in the street, and I appreciate that, you can’t not, with everyone offering, helping to take him out (…), If not, my son and I wouldn’t be able to go anywhere.”**“my sister supports me yes, and this year she has helped, when she sees that we are in real trouble, we can’t ask her for help. If she sees that we are really stuck, but I don’t want to tell her what I need.”**Impact on health**“I’m okay, well, We argue from time to time, because everyone gets upset,…, it’s not that I’m bitter, it’s just that we’re not going to agree on everything.”**“fine, fine. I can see that’s it’s more work now… I find it difficult to take the metro andro pull him. I don’t mean that I can’t pull him anymore, but I still do it.”*

## 4. Discussion

We studied changes in the personal support networks of informal caregivers that were selected on a purposive basis according to the objectives of the study over one year. Our findings showed a loss of both help with specific caregiving tasks and emotional support, as well as differences between men and women and between caregivers with low and high burden scores in the first wave. The greatest loss of support was observed among men with high scores. Those with low scores, by contrast, experienced no changes in personal network support. In the case of women, loss of support was more common in those with low burden scores.

In our study, male caregivers asked for and received more help when they needed it. When faced with an increase in burden, however, they were less used to reorganizing their caregiving duties, probably resulting in more negative coping strategies and a greater impact on their health. Women with low caregiver burden scores probably lost more support than men because they were less used to asking for help, largely because they felt well equipped to deal with adversities and were better at planning. This might explain why losing support from their network did not have a negative impact on their health. Because women accepted their caregiving role more readily than men, they demanded more of themselves when they had to shoulder more burden, possibly explaining why they experienced a greater deterioration in health than men following a loss of support. Women lost more emotional support than men but expressed more gratitude and appreciation for the support they received.

Our findings show that caregivers lost support over the year analyzed. A previous study found similar results, even over longer periods; the informal caregivers of people with dementia were more likely to have less contact with relatives, friends, and acquaintances when they had been providing care for at least 4 years [33]. We also observed that the selected men continued to receive more help with household chores than women, who, in addition, experienced a greater loss of help with physical mobility tasks. Our findings highlight the presence of traditional gender roles in caregiving. Other studies also highlight this fact. Men receive help with tasks that are socially considered feminine, while women are helped with tasks that are traditionally considered masculine [1,24].

We also observed differences in the reasons that were given for the non-involvement of male and female alters. Regardless of their relationship with the caregiver, women were perceived as having greater family responsibilities that prevented them from taking a more active role in caregiving. We also observed that daughters helped out more than sons. Another factor that emerged that was unrelated to gender differences but key to the organization of caregiving tasks was “propinquity,” which is a concept that some authors have defined as the geographic proximity of people who are connected [34]. An earlier cross-sectional study by our team found that men received more help from people living nearby than women [24]. Studies of the spatial distribution of personal networks and the scope of support received generated contrasting results in different study topics [35]. Our results suggested that geographic proximity was essential for providing help with specific caregiving tasks and in unforeseen circumstances, particularly in the case of more severely burdened caregivers and/or caregivers who were looking after someone with a higher level of dependency. This proximity was not a requisite for emotional support.

These results are consistent with the literature showing that support and interpersonal relationships [36,37,38] have a positive impact on health and caregiver burden [16,39]. There is also evidence showing that health is affected by coping strategies [40,41] and the way in which caregiver roles are assigned or negotiated [42].

The selected women in our study had a more positive attitude to caregiving than the men. They accepted their role as the primary caregiver more readily, as it was something they had largely expected to have to do at some stage in their lives. Accordingly, they tended to be better at managing and organizing their network resources and generally took a leading role for as long as their health permitted. When their circumstances changed (e.g., when their workload increased or health deteriorated), they were more adept than male caregivers at reshuffling resources and demanding more involvement from members of their network. Accordingly, our findings showed that a loss of support had less of an impact on the health of female caregivers who were in good initial health and who had a well-organized personal network. Women who perceived deterioration in their health expressed a very strong sense of duty toward care and mentioned that they had decided to take on this role after much deliberation. Nonetheless, their family biographies told of women who had served as primary caregivers from a very young age. Male caregivers, by contrast, found themselves thrust into the role of primary caregiver when there were no women in their immediate circles to take charge. Supporting previous reports [43], most of the men in our series were caring for their partners. Research has shown that men who become involved in caregiving may feel confused and unsure about how this new role fits with their identity as a man [44]. Data on their experiences and need for support, however, are still lacking due to the recent incorporation of men into the caregiving arena [45,46]. Female caregivers have worse self-perceived health than men [39,47,48], although, as indicated by stress and coping theories, men and women perceive, report, and cope with stressors differently. A study of caregiver burden in dementia care networks showed that women felt more stressed than men, but it also showed that they developed more personally [49]. Another study found that men who cared for their spouses were at higher risk of depression, while women caring for children or spouses had more positive caregiving experiences [39].

One strength of this study was the identical number of male and female caregivers analyzed and our coverage of a wide diversity of caregiving situations. Our findings, however, may be limited by the short interval between waves, as a year is probably insufficient to capture true changes in the care process. A longer follow-up time is needed to achieve more consistent results. It is important to note that we are living in a time of constant technological change and advances in diagnosis and treatment that are likely to prolong the care process and modify individual and social perceptions of health and disease.

Since caregivers were selected intentionally, and due to the type of quantitative analysis we carried out, our results are not conclusive, but we believe that they will contribute to deepening this area of study in future research. Personal network support is an undervalued resource. A greater understanding of personal networks is needed to inform policies that are aimed at building relational and social capital [50]. Such policies will have a key role in the reorganization and improvement of formal home care systems, whose importance has been brought to the fore by the current COVID-19 pandemic. It remains to be seen how this exceptional situation will affect informal caregiving in the years to come and how personal networks of male and female caregivers will change.

## 5. Conclusions

The selected male and female informal caregivers lost support from their personal networks over time. The losses were the greatest among men with high levels of burden and women with low levels of burden; furthermore, their reports and coping were different. Gender socialization equipped female caregivers in good health with better coping and organizational skills than their male counterparts. Men with a high caregiver burden and women with a strong sense of duty toward care experienced the greatest deterioration in self-perceived health. Our findings should be taken into account when allocating resources and designing actions to meet the needs of different profiles of caregivers. Interpersonal relationships are a resource that has not been taken into account to improve current care policies.

## Figures and Tables

**Table 1:** Sociodemographic and caregiving characteristics of participants.

		Level of Burden ^1^		Total
		None or Low	High	
		14 (43.8)	18 (56.2)	32 (100)
		*n* (%)		
Gender	Male	7 (21.9)	9 (28.1)	16 (50)
	Female	7 (21.9)	9 (28.1)	16 (50)
Time Providing Care (Years)	<3	3 (9.4)	5 (15.6)	8 (25)
	3–10	4 (12.5)	11 (34.4)	15 (46.9)
	>10	7 (21.9)	2 (6.3)	9 (28.1)
Age (Years)	≥65	5 (15.6)	9 (28.1)	14 (43.8)
	<65	9 (28.1)	9 (28.13)	18 (56.3)
Place of Residence	Rural	5 (15.6)	9 (28.2)	14 (43.8)
	Urban	9 (28.1)	9 (28.1)	18 (56.2)
Level of Education	No schooling or primary	6 (18.8)	8 (25.0)	14 (43.8)
	Secondary or tertiary level	8 (25.0)	10 (31.3)	18 (56.3)
Type of Relationship with Care Recipient	Man looking after partner	6 (18.8)	6 (18.8)	12 (37.5)
	Woman looking after partner	3 (9.4)	2 (6.3)	5 (15.6)
	Daughter looking after mother	3 (9.4)	5 (15.6)	8 (25.0)
	Son looking after mother	0 (0.0)	1 (3.1)	1 (3.1)
	Daughter looking after mother and father	0 (0.0)	2 (6.3)	2 (6.3)
	Son looking after mother and father and brother	0 (0.0)	2 (6.3)	2 (6.3)
	Mother looking after daughter	1 (3.1)	0 (0)	1 (3.1)
	Nephew looking after uncle	1 (3.1)	0 (0)	1 (3.1)

^1^ Zarit Burden Interview scores: <55, none or low burden; ≥56, high burden.

**Table 2:** Changes in support according to caregiver gender.

			First Wave		Second Wave		Difference	
		Caregiver (Ego)	Men	Women	Men	Women	Men	Women
		Total	*n* = 800 Alters		*n* = 800 Alters			
			*n* (%)					
Alters		No help from network members with specific caregiving tasks	311 (77.8)	312 (78.0)	320 (80.0)	319 (79.8)	9 (2.3)	7 (1.8)
	Help with Specific Caregiving Tasks	Personal care	33 (8.3)	44 (11.0)	27 (6.8)	37 (9.3)	−6 (−1.5)	−7 (−1.8)
		Physical mobility	43 (10.8)	45 (11.3)	40 (10.0)	40 (10.0)	−3 (−0.8)	−5 (−1.3)
		Household chores	37 (9.3)	31 (7.8)	36 (9.0)	31 (7.8)	−1 (−0.3)	0 (0.0)
		Nursing-type tasks	44 (11.0)	45 (11.3)	40 (10.0)	44 (11.0)	−4 (−1.0)	−1 (−0.3)
		Company keeping/ surveillance	56 (14.0)	62 (15.5)	50 (12.5)	54 (13.5)	−6 (−1.5)	−8 (−2.0)
		Activities outside the home	65 (16.3)	61 (15.3)	53 (13.3)	54 (13.5)	−12 (−3.0)	−7 (−1.8)
	Frequency of Help	Every day	21 (5.3)	24 (6.0)	17 (4.3)	29 (7.3)	−4 (−1.0)	5 (1.3)
		2 or 3 times a week	14 (3.5)	17 (4.3)	11 (2.8)	14 (3.5)	−3 (−0.8)	−3 (−0.8)
		Weekly	8 (2.0)	16 (4.0)	15 (3.8)	14 (3.5)	7 (1.8)	−2 (−0.5)
		Every 2 weeks	12 (3.0)	10 (2.5)	11 (2.8)	5 (1.3)	−1 (−0.3)	−5 (−1.3)
		Every month	21 (5.3)	4 (1.0)	14 (3.5)	7 (1.8)	−7 (−1.8)	3 (0.8)
		Every 2 or 3 months	5 (1.3)	8 (2.0)	6 (1.5)	6 (1.5)	1 (0.3)	−2 (−0.5)
		More than every 3 months	8 (2.0)	9 (2.3)	6 (1.5)	6 (1.5)	−2 (−0.5)	−3 (−0.8)
	Emotional Support	No	144 (36.0)	143 (35.8)	150 (37.5)	153 (38.3)	6 (1.5)	10 (2.5)
		Yes, for caregiving-related matters	21 (5.3)	31 (7.8)	37 (9.3)	42 (10.5)	16 (4.0)	11 (2.8)
		Yes, for non-caregiving-related matters	24 (6.0)	15 (3.8)	17 (4.3)	18 (4.5)	−7 (−1.8)	3 (0.8)
		Yes, for both of the above situations	211 (52.8)	211 (52.8)	196 (49.0)	187 (46.8)	−15 (−3.8)	−24 (−6.0)

**Table 3:** Changes in network support according to caregiver gender and burden.

			Male Caregiver (Ego)						Female Caregiver (Ego)					
			First Wave		Second Wave		Difference		First Wave		Second Wave		Difference	
			Level of Burden						Level of Burden					
			None or Low	High	None or Low	High	None or Low	High	None or Low	High	None or Low	High	None or Low	High
		Total	175 (100)	225 (100)	175 (100)	225 (100)			175 (100)	225 (100)	175 (100)	225 (100)		
Alters			*n* = 400 alters		*n* = 400 alters				*n* = 400 alters		*n* = 400 alters			
	Changes in help	No network members offering help	152 (86.9)	159 (70.7)	152 (86.9)	168 (74.7)	0 (0.0)	9 (4.0)	134 (76.6)	178 (79.1)	138 (78.9)	180 (80)	4 (2.3)	2 (0.9)
		Increase in help	4 (2.3)	12 (5.3)	2 (1.1)	24 (10.7)	−2 (−1.1)	12 (5.3)	11 (6.3)	10 (4.4)	12 (6.9)	19 (8.4)	1 (0.6)	9 (4.0)
		Decrease in help	0 (0.0)	4 (1.8)	1 (0.6)	8 (3.6)	1 (0.6)	4 (1.8)	2 (1.1)	12 (5.3)	6 (3.4)	11 (4.9)	4 (2.3)	−1 (−0.4)
		No change	19 (10.9)	50 (22.2)	20 (11.4)	25 (11.1)	1 (0.6)	−25 (−11.1)	28 (16.0)	25 (11.1)	19 (10.9)	15 (6.7)	−9 (−5.1)	−10 (−4.4)
	Emotional support	No	37 (21.1)	107 (47.6)	53 (30.3)	97 (43.1)	16 (9.1)	−10 (−4.4)	73 (41.7)	70 (31.1)	80 (45.7)	73 (32.4)	7 (4.0)	3 (1.3)
		Yes, for caregiving-related matters	9 (5.1)	12 (5.3)	13 (7.4)	24 (10.7)	4 (2.3)	12 (5.3)	6 (3.4)	25 (11.1)	13 (7.4)	29 (12.9)	7 (4.0)	4 (1.8)
		Yes, for non-caregiving-related matters	14 (8.0)	10 (4.4)	3 (1.7)	14 (6.2)	−11 (−6.3)	4 (1.8)	5 (2.9)	10 (4.4)	3 (1.7)	15 (6.70)	−2 (−1.1)	5 (2.2)
		Both situations	115 (65.7)	96 (42.7)	106 (60.6)	90 (40.0)	−9 (−5.1)	−6 (−2.7)	91 (52.0)	120 (53.3)	79 (45.1)	108 (48.0)	−12 (−6.9)	−12 (5.3)

## Data Availability

Data belong to the CUIDAR-SE Study and the data are owned by the Escuela Andaluza de Salud Pública and, hence, under the Escuela Andaluza‘s regulations, as it contains potentially sensitive information on individuals. People interested in data viewing can contact María del Mar García Calvente, the main researcher of the CUIDAR-SE Study. Escuela Andaluza de Salud Pública, Cuesta del Observatorio, 4, 18011 Granada, Spain, e-mail: mariadelmar.garcia.easp@juntadeandalucia.es.

## Appendix A

The following techniques were used to collect qualitative data.

Semi-structured interview script (first wave):

- Regarding the people you said do not help you, why do they not help you?
- If I had come a year ago, would you have mentioned the same people?
- Do you think that the support you have received has affected your health?

Semi-structured interview script (second wave):

- Why did you remove the people we have eliminated from your network?
- Of the people we have added, did they form part of your life last year? Why did you not mention them? Why have we included them this year?
- Of the people we have kept in the network, have there been any changes not noted so far that you think explain the changes in the support received this year?
- Why do the people who supported you last year no longer support you?
- Why do the people who did not support you last year not continue to do so?
- What circumstances have favored or hindered the changes in support received or not received from people in your personal network?
- How is your health this year? Do you think that your health has improved, got worse, or stayed the same as last year?
